# Increase in admission rates and symptom severity of childhood and adolescent anorexia nervosa in Europe during the COVID-19 pandemic: data from specialized eating disorder units in different European countries

**DOI:** 10.1186/s13034-022-00482-x

**Published:** 2022-06-20

**Authors:** Susanne Gilsbach, Maria Teresa Plana, Josefina Castro-Fornieles, Michela Gatta, Gunilla Paulson Karlsson, Itziar Flamarique, Jean-Philippe Raynaud, Anna Riva, Anne-Line Solberg, Annemarie A. van Elburg, Elisabet Wentz, Renata Nacinovich, Beate Herpertz-Dahlmann

**Affiliations:** 1 Department of Child and Adolescent Psychiatry, Psychosomatics, and Psychotherapy, RWTH Aachen University, Neuenhofer Weg 21, 52064 Aachen, Germany; 2 Child and Adolescent Psychiatry and Psychology Department, IDIBAPS, CIBERSAM, Institute Clínic of Neurosciences, Hospital Clínic of Barcelona, University of Barcelona, C. de Villarroel, 170, I2017SGR88108036 Barcelona, Spain; 3 Children and Adolescents Neuropsychiatry Unit, Woman and Child Health Department, University Hospital of Padova, University of Padova, Via VIII Febbraio, 2, 35122 Padua, PD Italy; 4 Eating Disorder Center Children & Young Adults, The Sahlgrenska University Hospital, University of Gothenburg, Vitaminvägen 17, 416 50 Göteborg, Sweden; 5 Service Universitaire de Psychiatrie de l’Enfant et de l’Adolescent, CHU de Toulouse, Hôpital Purpan, Université de Toulouse, Place du Docteur Baylac, 31059 Toulouse cedex 9, France; 6 Child and Adolescent Mental Health, San Gerardo Hospital, University of Milano Bicocca, Via G. B. Pergolesi, 33, 20900 Monza, MB Italy; 7 Faculty of Social Sciences, Utrecht University, Heidelberglaan 1, 3584 CS Utrecht, The Netherlands; 8 Centre for Eating Disorders, Altrecht Mental Health Institute, Rintveld, 3705WE Zeist, The Netherlands; 9 Department of Psychiatry and Neurochemistry, Institute of Neuroscience and Physiology, Sahlgrenska Academy, University of Gothenburg, Ätstörningsmottagning Högsbo, Sahlgrenska University Hospital, Lilla Kapplandsgatan 26B, 421 37 Västra Frölunda, Sweden

**Keywords:** COVID-19 pandemic, Anorexia nervosa, Hospital admissions, Europe

## Abstract

**Background:**

The COVID-19 pandemic, associated with confinement and social isolation, seems to have impacted the course of many mental disorders in children and adolescents. An increase in hospital admission rates for juvenile anorexia nervosa (AN) has been documented in many regions of the world. However, data from Europe are scarce.

**Methods:**

We asked clinicians in specialized eating disorder units in hospitals of maximum care in France, Germany, Italy, Spain, Sweden, and the Netherlands to report on (i) overall (inpatient and outpatient) and (ii) inpatient admission rates for adolescents with AN during 2019 and 2020. Additionally, a modified version of the COVID Isolation Eating Scale (CIES) was used to assess the child and adolescent psychiatrists’ estimations of a possible increase in symptom severity in children and adolescents with AN during the COVID-19 pandemic and to (iii) inquire about the contributing factors perceived by the caring professionals.

**Results:**

Four out of six representatives of European hospitals described a higher rate of overall admissions during the pandemic. Three hospitals out of six reported an increase in inpatient admissions, and two centres had constant high numbers of admissions of both outpatients and inpatients. The clinicians perceived a higher symptom severity in 2020 than in 2019, especially involving more frequent use of social media, longer duration of exercising, and more restrictive eating. They supposed an increase in social media consumption, a perceived “loss of control”, and a lack of in-person assessments and weight controls as the main contributing factors for the deterioration in AN numbers and symptomatology.

**Conclusions:**

The COVID-19 pandemic seems to have had a deep impact on symptom severity in AN, which is mirrored by a large increase in admission rates across Europe. An increase in exercise, social media consumption, a perceived “loss of control”, and a lack of face-to-face health care seem to have contributed to this development. Further investigation is required to identify which factors may lead to the increase in incidence and deterioration of childhood and adolescent AN. Possible preventive means for the future could include educating paediatricians and health care workers about AN, regular weight assessment, and home-based treatments.

## Background

The COVID-19 pandemic has contributed to an increase in the mental health problems of children and adolescents worldwide [1, 2]. An important impact has been observed in patients with preexisting eating disorders (EDs), particularly anorexia nervosa (AN) [2]. While many studies have focused on the effects of the COVID-19 pandemic on eating disorders in adults [2, 3], some more recent reports have targeted children and adolescents, who seem to be particularly vulnerable to the effects of the pandemic [1, 4]. Haripersad et al. [5] and Hansen et al. [6] reported a large increase in hospital admissions because of AN in adolescents during the COVID-19 pandemic in Western Australia and in a mixed urban–rural area in New Zealand, respectively. Similar trends for adolescents with AN were observed in Canada, with higher admission rates in regions with higher infection rates [7], and an Israelian study reported a 2.4-fold increase in admission rates of adolescents to the largest Israeli paediatric tertiary care hospital during the COVID-19 pandemic compared with the period 2015–2019 [8]. A study from the US demonstrated a large increase in admissions of 10- to 23-year-old patients with eating disorders (ED) during the first 12 months of the pandemic, which was more than double the mean number of admissions per year for the same timeframe for the previous 3 years [9].

In addition to an increase in prevalence, young patients with AN seem to be more severely ill than before the pandemic. In a cohort study comparing adolescents admitted with AN in 2020 to those admitted in 2019, the former had a lower body weight and higher rates of self-reported functional impairment as a result of ED symptoms and were more likely to be medically unstable [10]. Readmissions for acute medical complications of AN to a specialized ED unit in a paediatric medical centre in the US were 8 times higher within the first month after discharge during the COVID-19 pandemic than before [11].

The reasons for the increase in prevalence and/or severity of child and adolescent AN during the COVID-19 pandemic are not yet clear. Several contributing factors have been reported, such as school closures, social isolation, exacerbated comorbidities, and a cessation of face-to-face appointments [5]. A disruption of daily activities, increased social isolation, reduced access to the usual support networks, changes in physical activity, reduced access to health care services, and increased exposure to provoking messages on the internet negatively impacted ED symptoms in a UK survey with participants between 16 and 65 years of age [2]. A small qualitative Austrian study of adolescents with AN and their parents felt a negative impact of the interruption of treatment routines, restrictions on personal freedom, and more exposure to triggering situations related to AN symptoms due to the COVID-19 pandemic [12]. Similarly, adults reported an increase in body image concerns and restrictive eating [13] as well as in driven exercise and comorbid mental disorders during the COVID-19 pandemic [14]. In a study from the US in adults with a whole range of EDs, including AN, bulimia nervosa and ED not otherwise specified, the number of inpatients doubled, and the length of treatment increased significantly [15].

The goal of this study was to obtain data about the total number of patients admitted for AN and, more specifically, rates of inpatient admissions for AN across several European countries. Additionally, the study obtained qualitative data from mental health clinical experts using a modified standardized instrument to gather impressions about ED symptom severity.

## Methods

We asked well-known European researchers in the field of AN to designate representative specialized ED units for children and adolescents of hospitals of maximum care in their respective countries. In addition to our own unit, we asked six departments of child and adolescent psychiatry with specialized ED units in France, Italy, Sweden, and the Netherlands to participate. Data were collected by the administrative institution of the hospital regarding the total number of patients presenting with typical and atypical AN according to the DSM-5 [16] as well as inpatients admitted for AN in 2019 (1 January 2019–31 December 2019) and 2020 (1 January 2020–31 December 2020). Additionally, we asked for the mean age of the admitted patients, waiting time for treatment, and time frames of local lockdowns and school closures.

Furthermore, we requested ten mental health clinicians across the various European hospitals to give a subjective global impression of a possible change in symptom severity of the ED in the entire patient group they treated during the COVID-19 pandemic as well as their explanation for this change. The survey data were collected between March and May 2021.

There were two centres from Italy participating, but as we were not able to obtain exact patient numbers from the department of the University of Padua, we only used clinicians’ symptom severity estimation from the hospital of Padua as well as their evaluation of contributing factors.

### Assessment and measure

We modified the Isolation Eating Scale (CIES) [3] to ask the child and adolescent psychiatrists’ assessment of a possible increase in symptom severity in juvenile patients with AN during the COVID-19 pandemic. The original CIES is an internationally used self-report questionnaire with good psychometric properties that assesses the impact of confinement in adult and adolescent patients with ED and/or obesity (for a detailed description of the specific factor analyses please refer to 3; the psychometrics were established in a sample of 121 patients with mixed EDs and different ages; however, the majority of patients suffered fom AN). At the time of the investigation, questionnaires, and interviews to assess the impact of the COVID-19 pandemic on adolescent ED patients were rare. We selected 12 questions regarding concerns about body and weight, restrictive eating habits, physical exercise, hopeless and distressing thoughts about the current situation and the future, family members’ dieting, isolation, loneliness, nervousness, fears regarding COVID-19, and changes in social media use. Answers were given on a scale ranging from 0 = ‘not at all’ to 4 = ‘very strong’.

Furthermore, we listed seven possible explanations for an increase in prevalence and symptom severity of EDs (“loss of control”, “social media consumption”, “shame due to weight gain during the first lockdown”, “depression and loneliness”, “boredom and frustration”, “reduced personal medical assessment and weight control” and “other”) and let the clinicians rate their significance (“no impact”, “medium impact”, “high impact”).

Finally, we asked the clinicians to provide their subjective rating of which of the national lockdowns contributed to the highest increase in AN admissions.

As explained above, the clinicians rated their qualitative impression of the total group of patients they treated, not of individual patients.

### Participating clinicians

Symptom severity was assessed by eight medical doctors (MTP, MG, IF, JPR, AR, AAvE, RN, and BHD), one clinical psychologist (ALS), and one family therapist and social worker (GPK).

### Statistical analyses

We plotted the absolute number of admissions for AN provided by the respective centres for 2019 and 2020, depicting the total number of admissions and the number of inpatient admissions, respectively. In addition, we reported the indicated mean age of the total number of admissions for AN for each single treatment centre in 2019 and 2020.

The total number of weeks in lockdown and of school closure were summed up. However, the exact dates of lockdowns and school closures varied even between different regions of an individual country. Moreover, there were different stages of restrictions within the single lockdowns, and school closures differed regarding stages of intensity as well as different age groups, e.g., in some countries, the school closures were limited to children or younger adolescents. Therefore, the given time spans are only approximations.

The clinicians’ subjective assessment of the ED-associated increase in symptom severity was summarized for all countries and averaged for each respective item.

Finally, we plotted the impact of possible contributing factors based on the estimation of the participating clinicians.

## Results

**Fig. 1:**
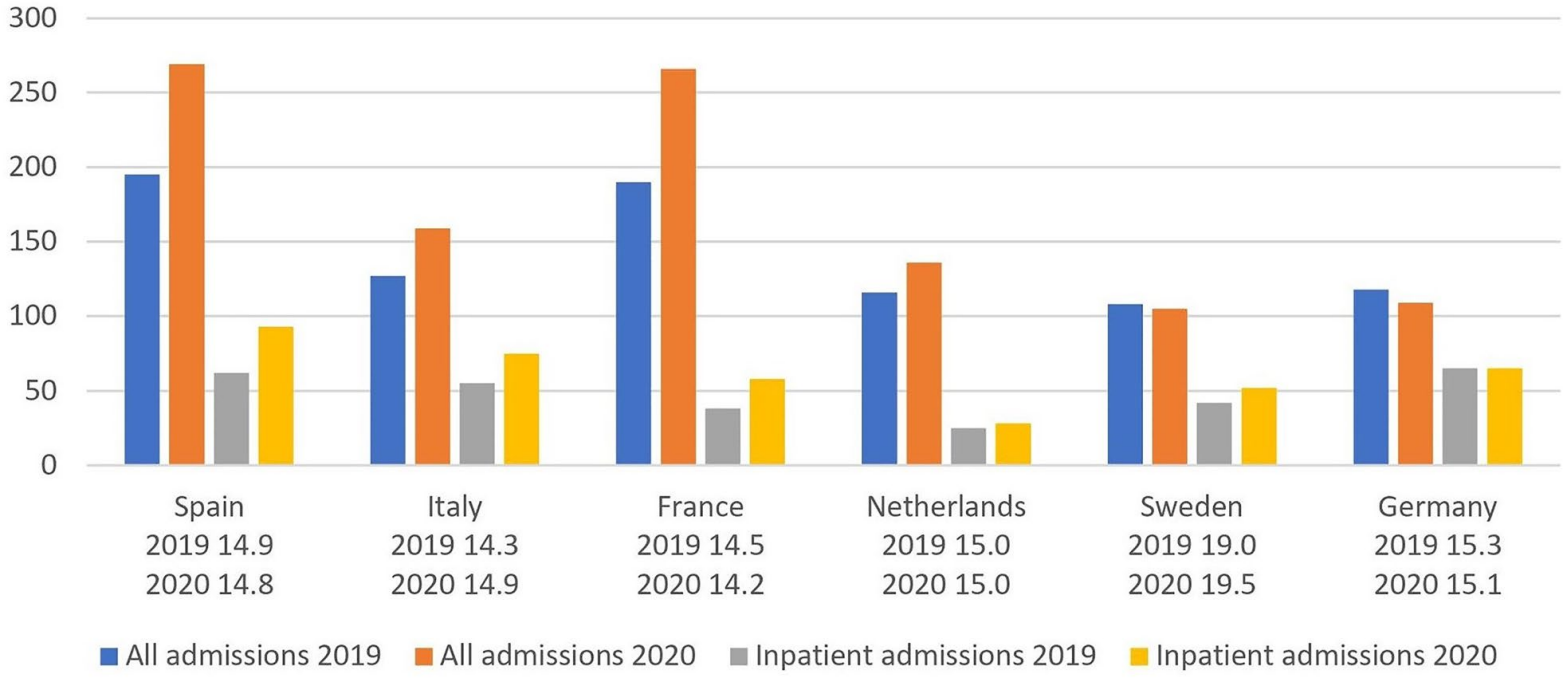
Number of hospital admissions and patients` mean age in 2019 and 2020

In four out of six ED centres, there was an increase in total (outpatient and inpatient) admissions. There was also a substantial increase in inpatient admissions in three out of six centres (see Fig. 1).

The Dutch centre had only an overall increase in admissions, whereas the number of inpatients remained stable. The Swedish and the German centres had an approximately unchanged number of patients, both in terms of total patients and inpatients.

All ED departments witnessed the greatest increase in admissions at the beginning of the second lockdown. Between the time before and during the lockdowns, there was no significant change in age at the time of admission in any participating department.

With the exception of the Netherlands, waiting times for hospital admissions increased in five out of six centres (in Sweden from 20 to 52 days, in Monza, Italy, from 11 to 25 days, in Padova, Italy, from 45 to 90 days, in France from 21 to 41 days and in Germany from 70 to 112 days). In the Netherlands, waiting time during the lockdowns was even shorter because the respective department started a “crisis management” to care for the most acute patients. Five out of seven centres had a longer average length of stay. The departments in Germany and Sweden reported no change in the duration of hospital treatment.

Apart from Sweden, there were two lockdowns in all countries included in this study until May 2021. Except for the Netherlands, the periods of the lockdowns and the school closures corresponded in all selected countries. In the Netherlands, lockdowns and school closures lasted approximately 8 to 10 weeks longer than in the other European regions (see Table 1).

**Table 1:** Number and duration of lockdown and school closures until the end of 2020

	Number of lockdowns	Number of weeks in lockdown (in total)	Number of weeks of school closures (in total)
Spain	2	14	14
Italy	2	14	17
France	2	15	14
Netherlands	2	23	20
Germany	2	13	13
Sweden	0	0	0

The numbers are approximations. Note, however, that both lockdowns and school closures varied in intensity depending on the age group and the respective country

**Fig. 2:**
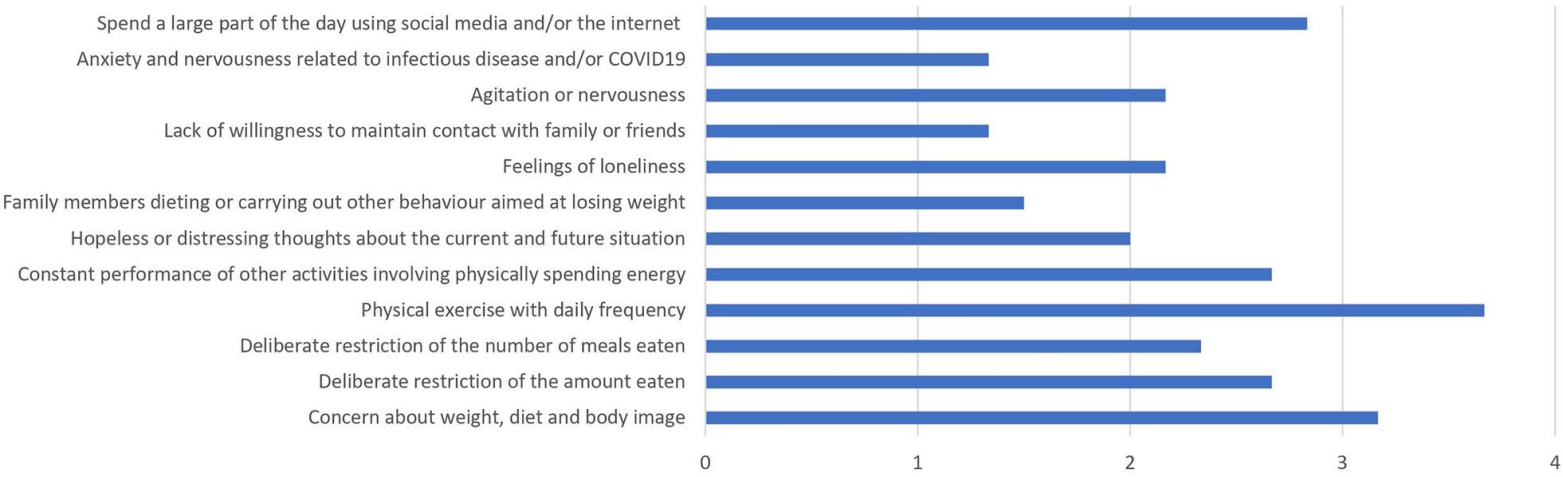
Mean increase of COVID19-associated alterations influencing symptoms of AN according to the subjective evaluation of 10 European clinicians

The reported increase in AN symptomology estimated by the abovementioned 10 clinicians revealed subjectively perceived aggravation in all domains during the COVID-19 pandemic, as displayed in Fig. 2.

Among all domains, the clinicians described the highest subjectively experienced increase in the amount of daily practised physical exercise, followed by concerns about weight, diet and body image, and social media use.

**Fig. 3:**
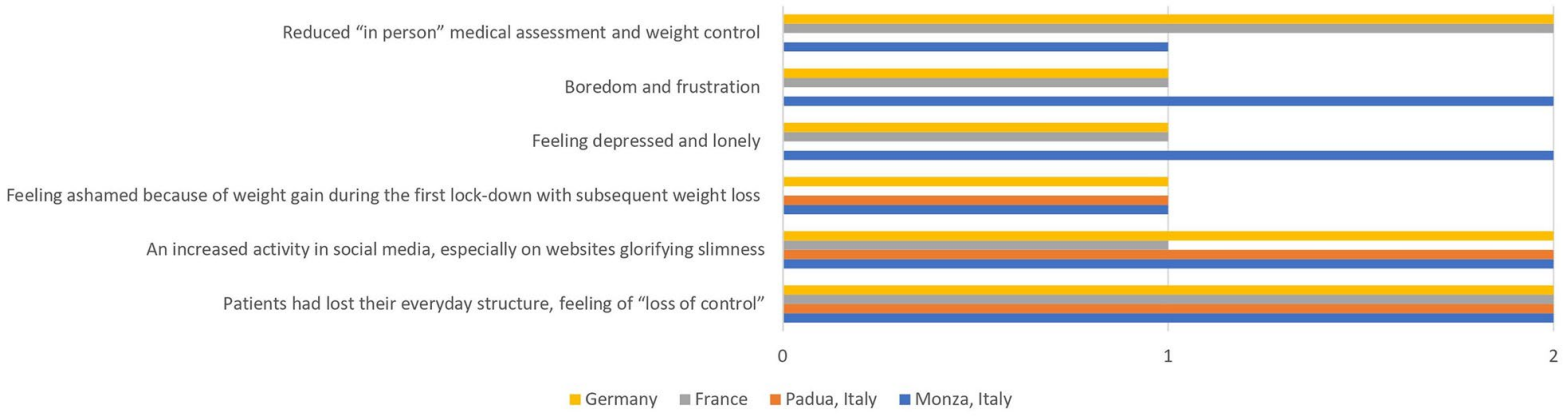
Perceived impact of contributing factors on COVID-19-associated incidence and severity of AN. Legend: O = no impact, 1 = medium impact, 2 = high impact

Based on the clinicians´ subjective evaluation, the following figure demonstrates the contributing factors for the higher COVID-19-associated hospitalization rate (see Fig. 3).

## Discussion

Our results confirm an increase in hospital admissions to specialized ED units in four out of six northern, central, and southern European countries. The undetectable increase in the department in Germany is due to a ceiling effect because only a limited number of patients with AN could be admitted, which had already been reached in 2019 and could not be increased further in 2020. The overall number of patients in the Swedish centre did not increase; there was only a small rise in the number of inpatients. The reason for this is not clear. One explanation might be the lack of lockdowns and school closures in Sweden leading to a less intense disruption of everyday life. Note, however, that the patient group treated in the Swedish centre was considerably older than the patients treated in the other centres. Most likely, in the adolescent samples of the other countries, the parents decided to bring their daughter/son earlier into hospital, while the older patients—apart from the very sick who urgently needed inpatient treatment—protracted a more intensive treatment.

The duration of waiting time nearly doubled in all countries except for the Netherlands. However, we do not know whether this was due to the increase in patients needing hospitalization or to a worsening of the disorder resulting in longer hospital stays.

Corresponding to the latter statement, clinicians experienced in the treatment of ED estimated the symptom severity of AN to be more severe than before the pandemic, thus confirming the development seen in other Western countries, such as Australia and Canada [7, 17]. Looking more specifically at the various domains of eating disordered behaviour, we noted the highest increase in using social media and exercising. In the work of Philippou et al. [17] and Branley-Bell and Talbot [2], an increase in exercise was also one of the most striking changes, as well as an increase in restrictive eating, which we also observed in our study.

There are several possible explanations for the rise in ED symptoms, as well as of general psychopathology. Among other problems, individuals complained of an interruption in regular sports activities, too much spare time, more exposure to triggering social media, a lack of social contacts with a negative impact on mood, and a reduction in treatment offers. Many patients reported reduced feelings of control. During the lockdowns in the different regions, nearly all family members stayed at home, and the usual daily structure, including regular meals at fixed times, was lost. Moreover, the COVID-19-associated constraints often limited access to regular physical activity, which, in combination with unregular meals, probably intensified weight and body shape concerns [18]. Strikingly, a higher perception of general external control was associated with a greater probability of recovery [19]. In line with our European assessment of the supposed pandemic-induced aggravation of AN symptomatology in adolescents, Schlegl et al. [20] conducted a survey with German adolescent and adult patients discharged from inpatient treatment in 2019 and found an increase in eating, body shape and weight concerns, a higher drive for physical activity, loneliness, sadness, and inner restlessness during the pandemic, and a decrease of access to in-person psychotherapists and visits to general practitioners of 37% and 46%, respectively. These findings are very much in line with our own results, e.g., the experts’ subjective estimations of contributing factors for the increase in admissions and symptom severity.

The present study has several limitations. First, health care systems and admission policies, especially for AN, in the countries involved are very different; for example, in Germany, inpatient treatment is still the gold standard in the treatment of adolescent AN, while in the Netherlands and Scandinavia, many more patients are treated on an outpatient basis. In addition, we had no data on mean admission BMI, the duration of illness, psychiatric comorbidity, or sociodemographic data. This was a retrospective study in which not all demographic and clinical parameters had been assessed in detail by the different institutions. However, it seemed important for us to obtain an overview of the development of the morbidity and severity of AN in several European countries at the peak of the pandemic. Second, because the study had not been planned before the pandemic, we had no baseline data on symptom severity prior to the pandemic, so the pronounced increase in eating disorders and general psychopathologies (e.g., depression, anxiety) is only based on the subjective impression of the clinicians during the pandemic. Most likely, there was also a discrepancy in illness severity of the admitted patients in the European hospitals enumerated above, which makes a comparison between countries very difficult. Third, we assessed only the number of cases admitted for AN and not the number of persons. Therefore, in our study, we could not determine whether the pandemic contributed to a higher new occurrence of AN or whether it was associated with a higher rate of relapse.

In conclusion, the COVID-19 pandemic seems to have had a deep impact on the burden of AN, which is mirrored by a mostly large increase in admission rates across Europe. In comparison to other mental disorders, symptom worsening was stated most frequently by patients with AN [21]. They are a particularly vulnerable group during a pandemic with contact restrictions because of their tendency towards social isolation, the danger of weight loss, and the high number of medical complications associated with AN [10]. In addition, the loss of the usual daily structure often associated with a feeling of loss of control might have reinforced typical symptoms of AN, such as weight phobia and body shape concerns. The overall recommendation to intensify teleconsulting and telepsychotherapy for patients with mental illness might not be fully suitable for these patients [22] because an assessment of weight is no longer guaranteed. Helpful alternatives could involve better information for paediatricians and health care workers to be aware of a developing ED during a pandemic and to include regular weight control in routine visits to the paediatrician or general practitioner, such as regular medical checks, as legalized in Germany at the ages of 12–14 and 16–17 years. In addition, there is growing evidence for the effect of home-based treatment for childhood and adolescent AN [23, 24]. More research is needed to determine which pandemic-associated factors contribute to the development and burden of eating disorders, especially in young people, and how to offer rapid support.

## Data Availability

The datasets used and/or analyzed during the current study are available from the corresponding author on reasonable request.

## References

[1] Ravens-Sieberer U, Kaman A, Erhart M, Devine J, Schlack R, Otto C (2021). Impact of the COVID-19 pandemic on quality of life and mental health in children and adolescents in Germany. Eur Child Adolesc Psychiatry.

[2] Branley-Bell D, Talbot CV (2020). Exploring the impact of the COVID-19 pandemic and UK lockdown on individuals with experience of eating disorders. J Eat Disord.

[3] Fernández-Aranda F, Munguía L, Mestre-Bach G, Steward T, Etxandi M, Baenas I, et al. COVID Isolation Eating Scale (CIES): Analysis of the impact of confinement in eating disorders and obesity—a collaborative international study. In: European Eating Disorders Review. John Wiley and Sons Ltd; 2020. p. 871–83. 10.1002/erv.2784 PMC7537123 32954595

[4] Singh S, Roy D, Sinha K, Parveen S, Sharma G, Joshi G (2020). Impact of COVID-19 and lockdown on mental health of children and adolescents: a narrative review with recommendations. Psychiatry Res.

[5] Haripersad YV, Kannegiesser-Bailey M, Morton K, Skeldon S, Shipton N, Edwards K (2021). Outbreak of anorexia nervosa admissions during the COVID-19 pandemic. Arch Dis Childhood..

[6] Hansen SJ, Stephan A, Menkes DB (2021). The impact of COVID-19 on eating disorder referrals and admissions in Waikato, New Zealand. J Eat Disord.

[7] Agostino H, Burstein B, Moubayed D, Taddeo D, Grady R, Vyver E (2021). Trends in the incidence of new-onset anorexia nervosa and atypical anorexia nervosa among youth during the COVID-19 pandemic in Canada. JAMA Netw Open.

[8] Goldberg L, Ziv A, Vardi Y, Hadas S, Zuabi T, Yeshareem L (2022). The effect of COVID-19 pandemic on hospitalizations and disease characteristics of adolescents with anorexia nervosa. Eur J Pediatr.

[9] Otto AK, Jary JM, Sturza J, Miller CA, Prohaska N, Bravender T (2021). Medical admissions among adolescents with eating disorders during the COVID-19 Pandemic. Pediatrics.

[10] Spettigue W, Obeid N, Erbach M, Feder S, Finner N, Harrison ME (2021). The impact of COVID-19 on adolescents with eating disorders: a cohort study. J Eat Disord.

[11] Matthews A, Kramer RA, Peterson CM, Mitan L (2021). Higher admission and rapid readmission rates among medically hospitalized youth with anorexia nervosa/atypical anorexia nervosa during COVID-19. Eat Behav.

[12] Zeiler M, Wittek T, Kahlenberg L, Gröbner EM, Nitsch M, Wagner G (2021). Impact of covid-19 confinement on adolescent patients with anorexia nervosa: a qualitative interview study involving adolescents and parents. Int J Environ Res Public Health.

[13] Mehta K (2021). To what extent does the COVID-19 pandemic impact patients with anorexia nervosa?. BJPsych Open.

[14] Miskovic-Wheatley J, Koreshe E, Kim M, Simeone R, Maguire S (2022). The impact of the COVID-19 pandemic and associated public health response on people with eating disorder symptomatology: an Australian study. J Eat Disord.

[15] Asch DA, Buresh J, Allison KC, Islam N, Sheils NE, Doshi JA (2021). Trends in US patients receiving care for eating disorders and other common behavioral health conditions before and during the COVID-19 pandemic. JAMA Netw Open.

[16] APA. American Psychiatric Association, 2013. Diagnostic and statistical manual of mental disorders (5th ed.). American Journal of Psychiatry. 2013.

[17] Phillipou A, Meyer D, Neill E, Tan EJ, Toh WL, Van Rheenen TE (2020). Eating and exercise behaviors in eating disorders and the general population during the COVID-19 pandemic in Australia: Initial results from the COLLATE project. Int J Eat Disord.

[18] Rodgers RF, Lombardo C, Cerolini S, Franko DL, Omori M, Fuller-Tyszkiewicz M (2020). The impact of the COVID-19 pandemic on eating disorder risk and symptoms. Int J Eat Disord.

[19] Branley-Bell D, Talbot CV (2021). “It is the only constant in what feels like a completely upside down and scary world”: Living with an eating disorder during COVID-19 and the importance of perceived control for recovery and relapse. Appetite.

[20] Schlegl S, Maier J, Meule A, Voderholzer U (2020). Eating disorders in times of the COVID-19 pandemic—results from an online survey of patients with anorexia nervosa. Int J Eat Disord.

[21] Favreau M, Hillert A, Osen B, Gärtner T, Hunatschek S, Riese M (2021). Psychological consequences and differential impact of the COVID-19 pandemic in patients with mental disorders. Psychiatry Res.

[22] Couturier J, Pellegrini D, Miller C, Bhatnagar N, Boachie A, Bourret K (2021). The COVID-19 pandemic and eating disorders in children, adolescents, and emerging adults: virtual care recommendations from the Canadian consensus panel during COVID-19 and beyond. J Eat Disord.

[23] Herpertz-Dahlmann B, Borzikowsky C, Altdorf S, Heider K, Dempfle A, Dahmen B (2021). ‘Therapists in action’—Home treatment in adolescent anorexia nervosa: A stepped care approach to shorten inpatient treatment. Eur Eat Disord Rev.

[24] Flütsch N, Hilti N, Schräer C, Soumana M, Probst F, Häberling I (2021). Feasibility and acceptability of home treatment as an add-on to family based therapy for adolescents with anorexia nervosa. A case series. Int J Eat Disord.

